# Supplementary material for: BERNN: Enhancing classification of Liquid Chromatography Mass Spectrometry data with batch effect removal neural networks
Source: Nat Commun. 2024 May 6;15:3777. doi: 10.1038/s41467-024-48177-5 (PMC11074280; doi:10.1038/s41467-024-48177-5)
Supplement: Supplementary file 1 — Supplementary Information [file 41467_2024_48177_MOESM1_ESM.pdf]

Supplementary

Supplementary Figures

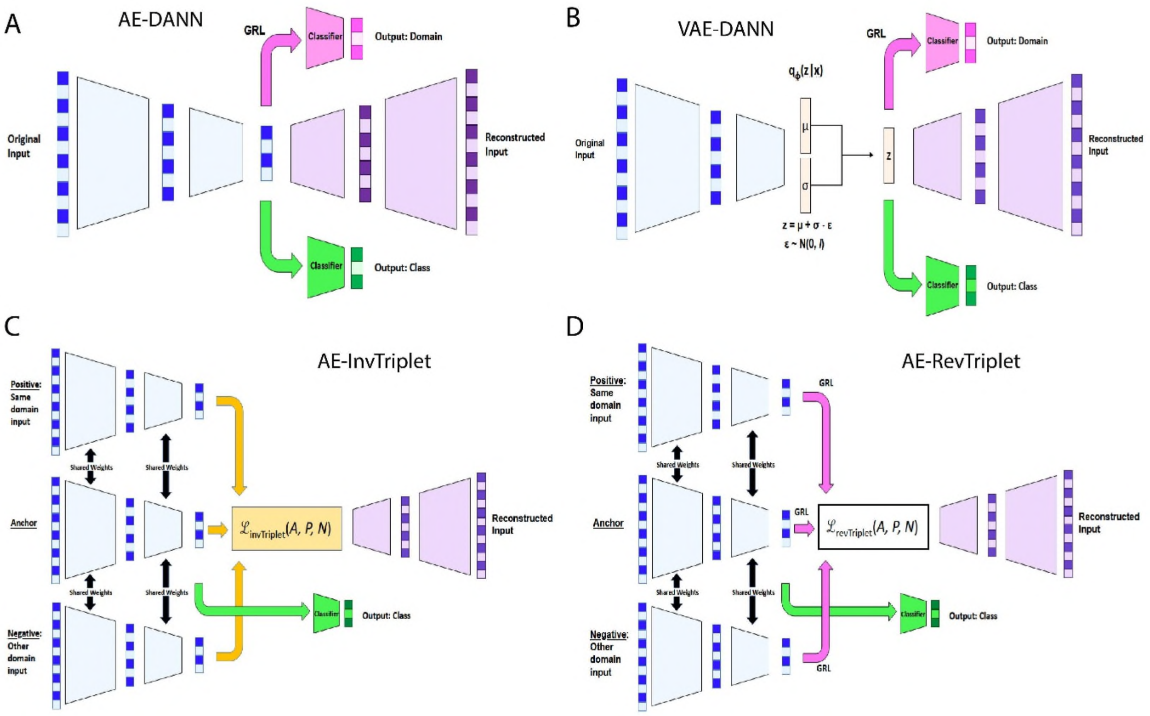

Supplementary Figure 1: Complete models architectures.

A) AE-DANN is an autoencoder with a domain classifier trained with a gradient reversal layer (GRL). B) VAE-DANN is a variational autoencoder with a domain classifier trained with a gradient reversal layer (GRL), where ϕ represents the parameters of the encoder (q), the parameters learned by the encoder are μ, σ and ε, which correspond to the mean, variance and gaussian noise, respectively. C) AE-InvTriplet is an Autoencoder that uses the inverse triplet loss to make the new representation batch-free. The encoders for the positive, negative and anchor samples all share the same weights. D) AE-RevTriplet is an Autoencoder that uses the reverse triplet loss to make the new representation batch-free. The encoders for the positive, negative and anchor samples all share the same weights. For all models, the autoencoder and methods to remove batch effects (DANN, invTriplet or revTriplet), if used, are trained during warmup. The label classifier is trained either with the rest of the model frozen (Alzheimer) or not (Adenocarcinoma and AgingMice). Not all models are depicted, but all models can be made from a combination of these models. AE is like A but without the domain classifier. VAE is like B but without the domain classifier. VAE-invTriplet is like C but the bottleneck encodes a gaussian distribution like in B. VAE-revTriplet is like D, but the bottleneck encodes a gaussian distribution like in B. The models have many modules in common, which are depicted using distinct colors.

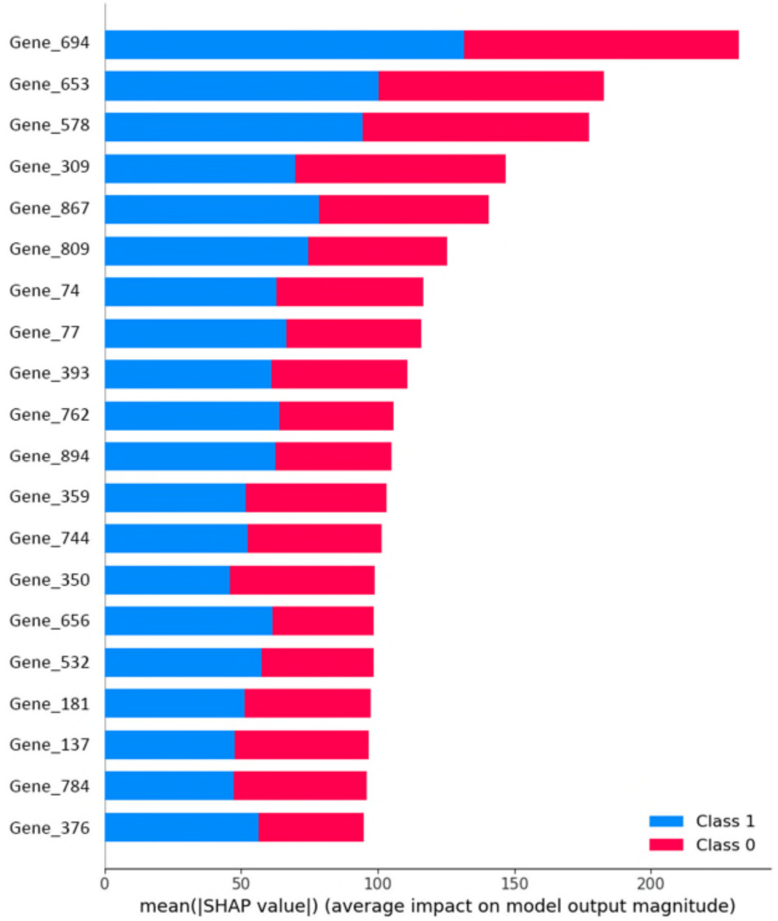

Supplementary Figure 2: Deep Learning model interpretation using SHAP's DeepExplainer.

Depicted here are the top 20 genes associated with the best classifier for the Alzheimer dataset, VAE-DANN. The higher the mean absolute SHAP values, the higher its importance to explain the model's decision. Class 0 is control and class 1 is Alzheimer's disease with dementia (DEM-AD).

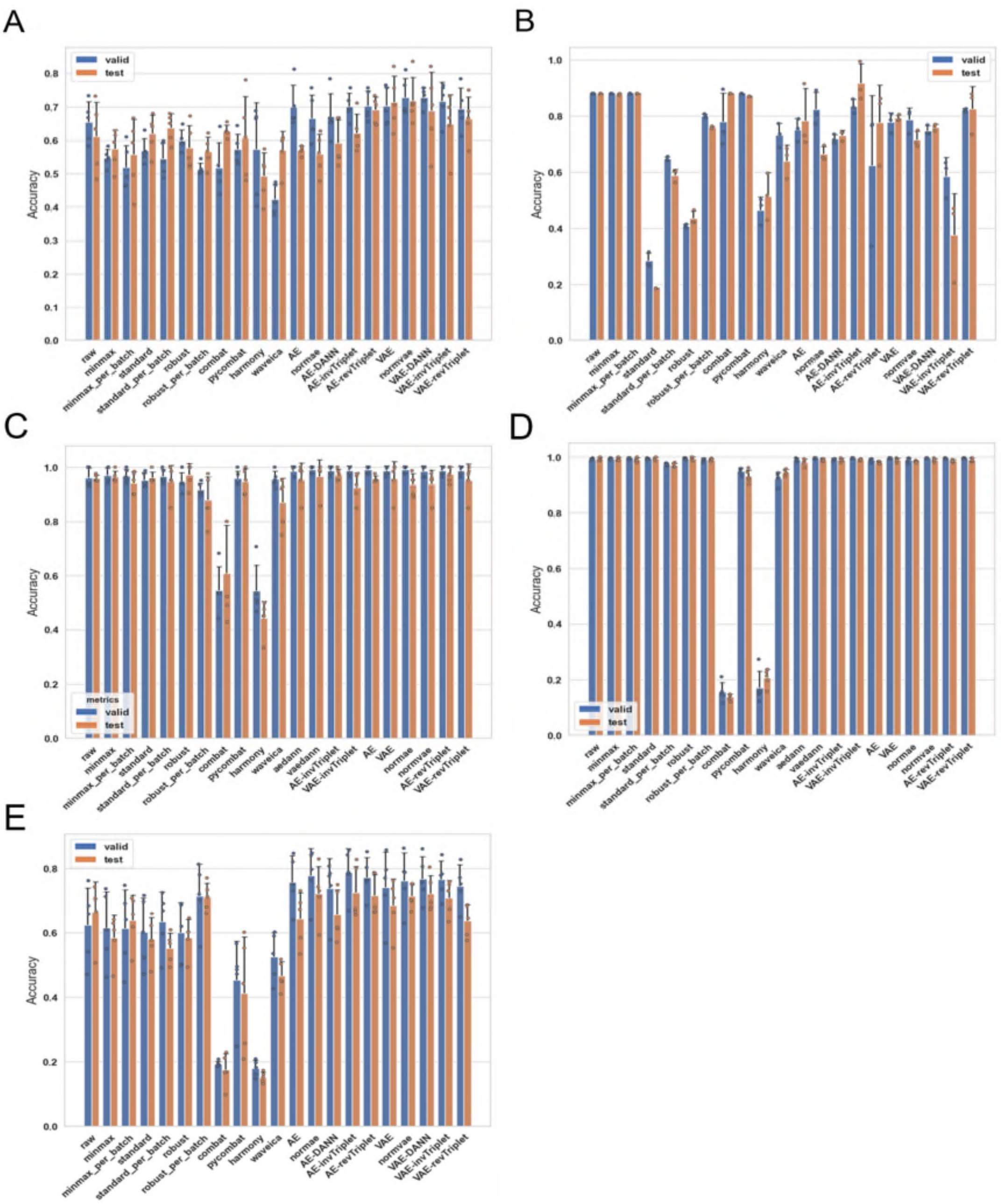

Supplementary Figure 3: Accuracies of the three datasets studied.

A) Alzheimer's disease dataset, B) Adenocarcinoma dataset, C) AgingMice dataset, D) Benchmark dataset and E) Mixed tissues dataset. (A, C, D, E) Error bars are derived from the results of 5-fold cross-validation (n=5). (B) Error bars are derived from the results of 3-fold cross-validation (n=3). Source data are provided as a Source Data file.

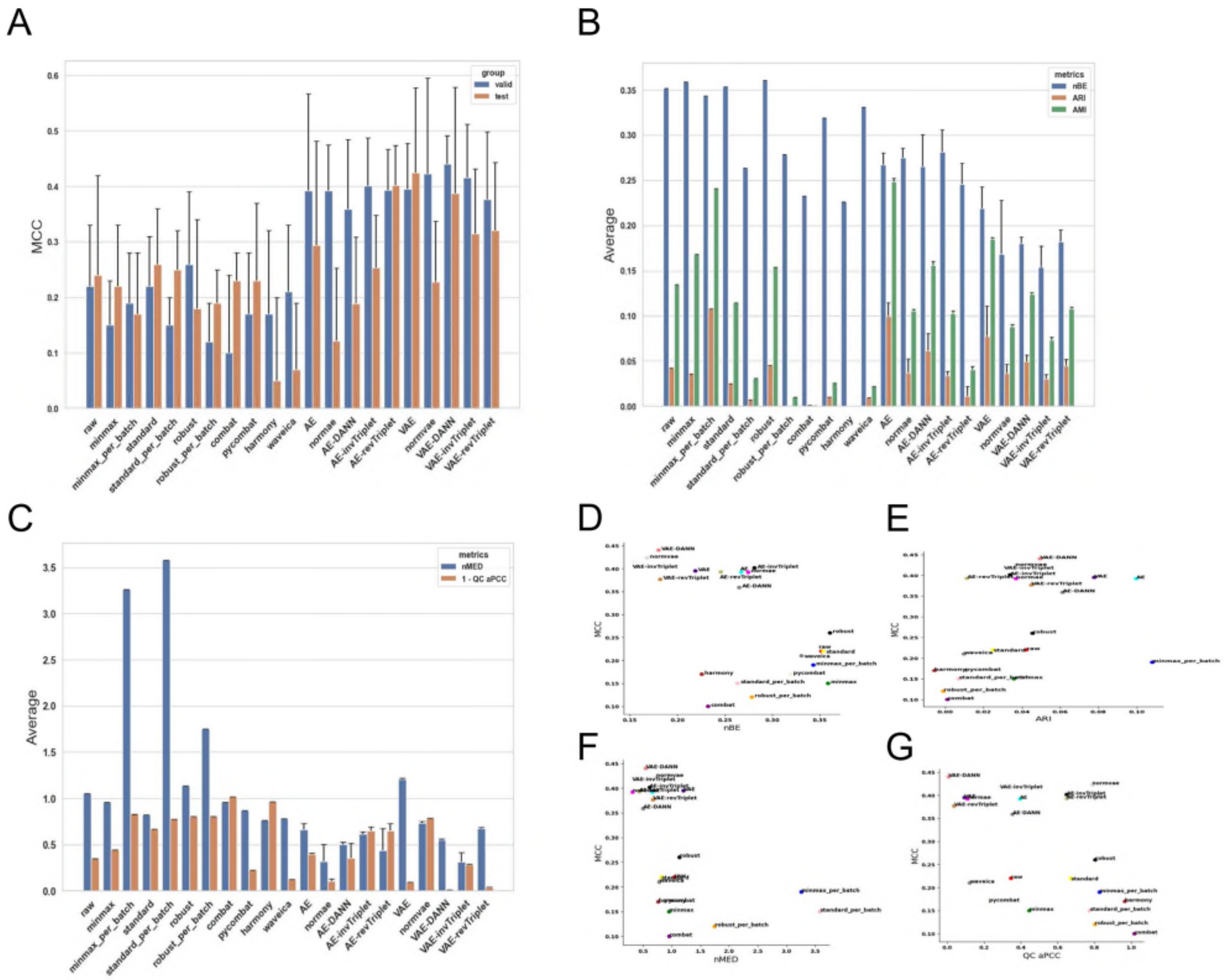

Supplementary Figure 4: Complete metrics on the Alzheimer dataset.

A) Valid and test MCC (Matthews Correlation Coefficient) scores for all methods benchmarked. Higher is better. B) Batch mixing metrics: normalized Batch Entropy (nBE), Adjusted Rand Index (ARI), Adjusted Mutual Information (AMI). The maximum entropy is ln(21). Smaller is better. C) QC metrics: Normalized Median Euclidean distance (nMED) and QC average Pearson Correlation Coefficient (qc_aPCC). Lower nMED and 1-qc_aPCC is better. (D-G) MCC is compared to D) nBE, E) ARI, F) QC nMED and G) QC aPCC. Error bars are derived from the results of 5-fold cross-validation (n=5). Source data are provided as a Source Data file.

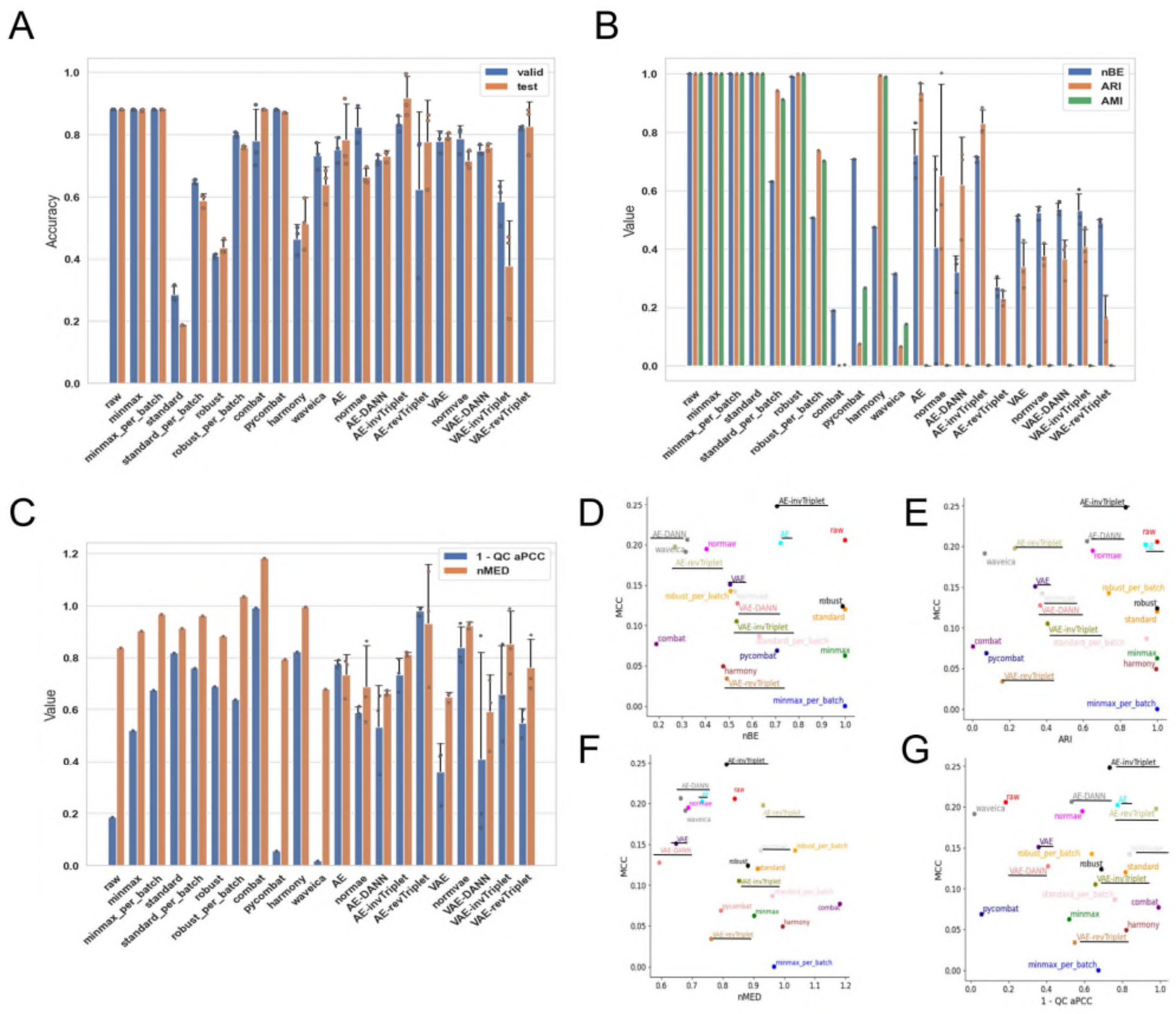

Supplementary Figure 5: Complete metrics on the Adenocarcinoma dataset.

A) Valid and test MCC scores for all methods benchmarked. Higher is better. B) Batch mixing metrics: normalized Batch Entropy (nBE), Adjusted Rand Index (ARI), Adjusted Mutual Information (AMI). The maximum entropy is ln(3). Smaller is better. C) QC metrics: Normalized Median Euclidean distance (QC nMED) and QC average Pearson Correlation Coefficient (qc_aPCC). Lower nMED and 1-qc_aPCC is better. (D-G) MCC is compared to D) nBE, E) ARI, F) QC nMED and G) QC aPCC. Error bars are derived from the results of 3-fold cross-validation (n=3). Source data are provided as a Source Data file.

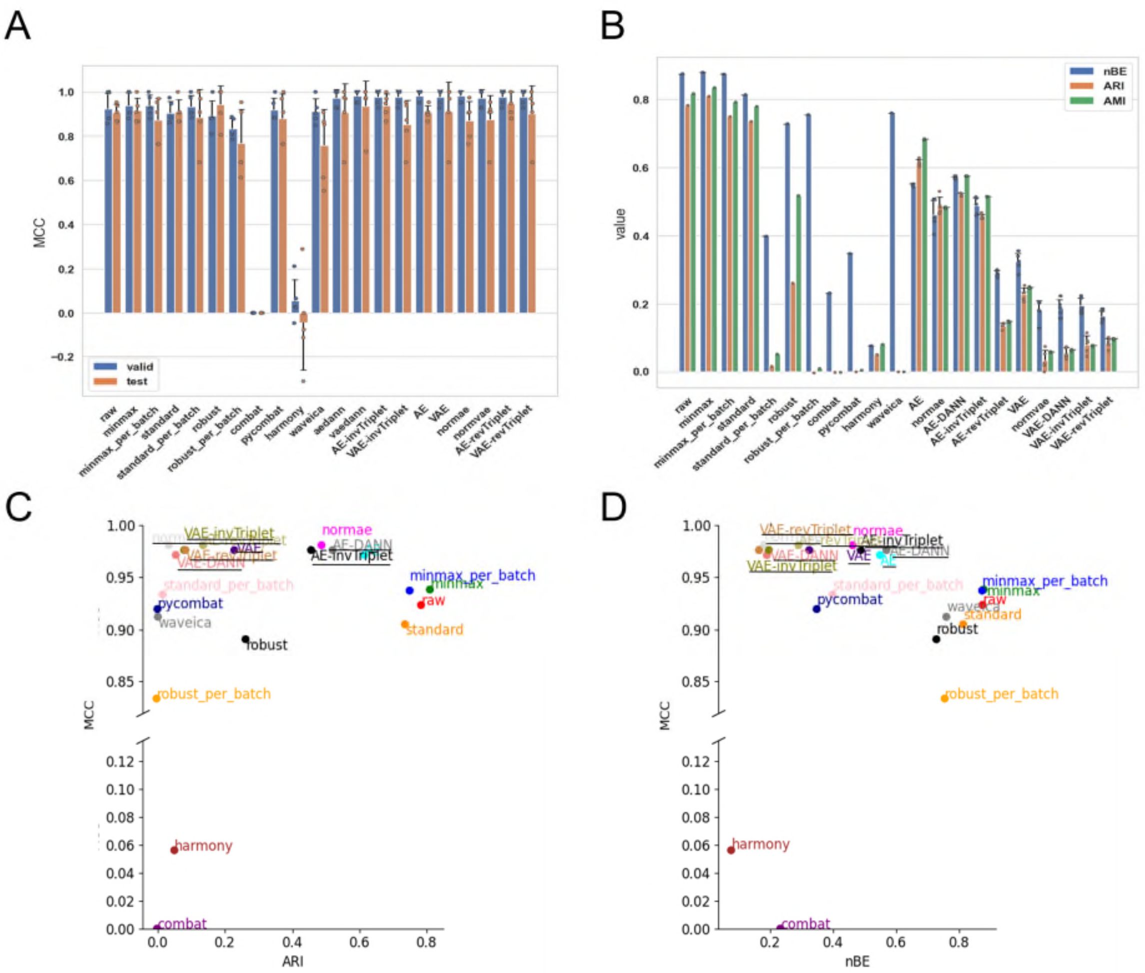

Supplementary Figure 6: Complete metrics on the AgingMice dataset.

A) Valid and test MCC scores for all methods benchmarked. Higher is better. B) Batch mixing metrics: normalized Batch Entropy (nBE), Adjusted Rand Index (ARI), Adjusted Mutual Information (AMI). The maximum entropy is ln(7). Smaller is better. (C-D) MCC is compared to C) nBE and D) ARI. Error bars are derived from the results of 5-fold cross-validation (n=5). Source data are provided as a Source Data file.

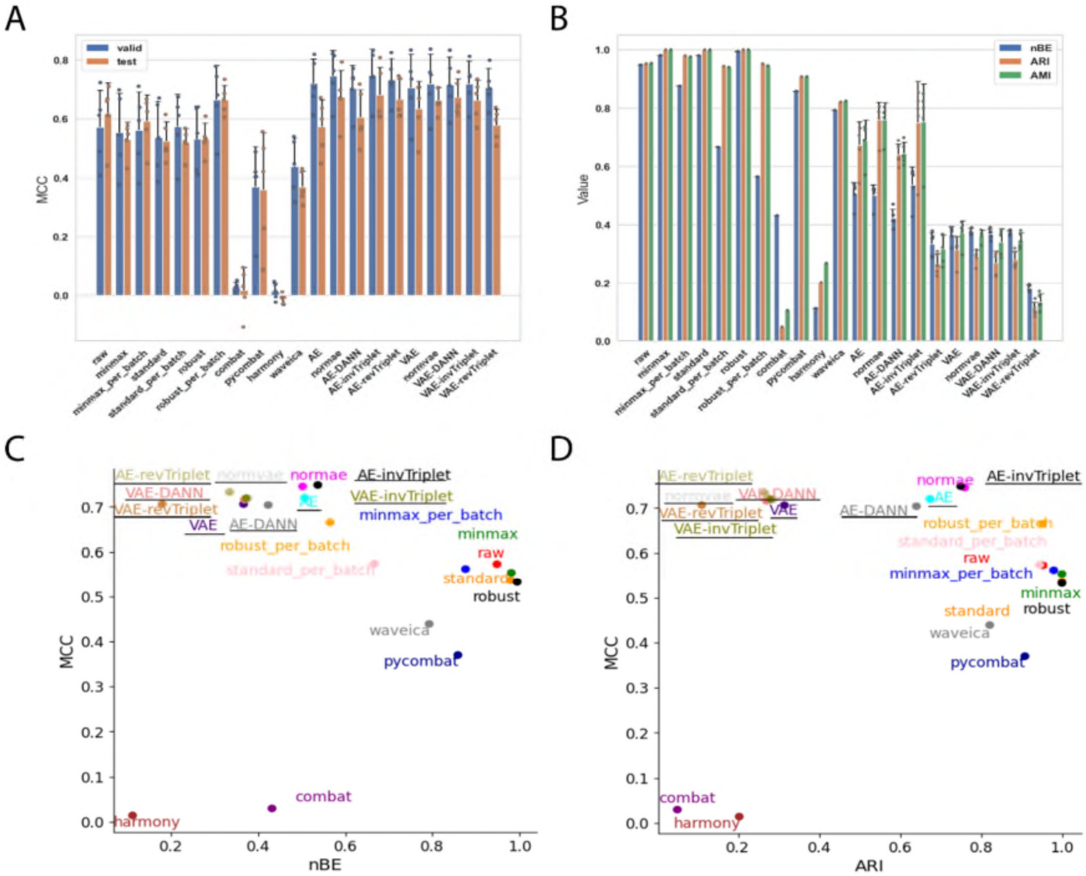

Supplementary Figure 7: Complete metrics on the Benchmark dataset.

A) Valid and test MCC scores for all methods benchmarked. Higher is better. B) Batch mixing metrics: normalized Batch Entropy (nBE), Adjusted Rand Index (ARI), Adjusted Mutual Information (AMI). The maximum entropy is ln(7). Smaller is better. (C-D) MCC is compared to C) nBE and D) ARI. Error bars are derived from the results of 5-fold cross-validation (n=5). Source data are provided as a Source Data file.

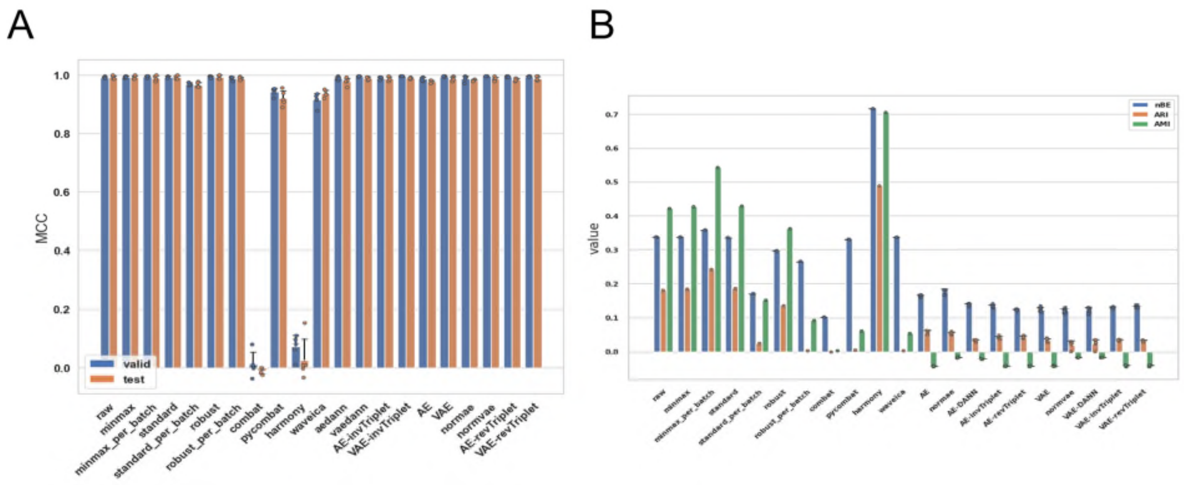

Supplementary Figure 8: Complete metrics on the Mixed tissues dataset.

A) Valid and test MCC scores for all methods benchmarked. Higher is better. B) Batch mixing metrics: normalized Batch Entropy (nBE), Adjusted Rand Index (ARI), Adjusted Mutual Information (AMI). The maximum entropy is ln(78). Smaller is better. Error bars are derived from the results of 5-fold cross-validation (n=5). Source data are provided as a Source Data file.

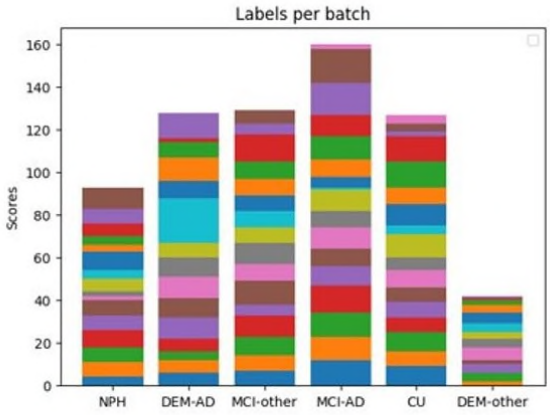

Supplementary Figure 9: Number of labels per batch for all 6 categories composing complete dataset.

Samples from all categories are used to train the autoencoder and batch classifier, but the labels classifier only learn to distinguish DEM-AD and CU. NPH: Normal Pressure Hydranencephalies, DEM-AD: Dementia from Alzheimer's Disease, MCI-other: Mild Cognitive Impairment from another disease than Alzheimer's, MCI-AD: Mild Cognitive Impairment from Alzheimer's Disease, CU: Cognitively Unimpaired, DEM-other: Dementia from another disease than Alzheimer's.

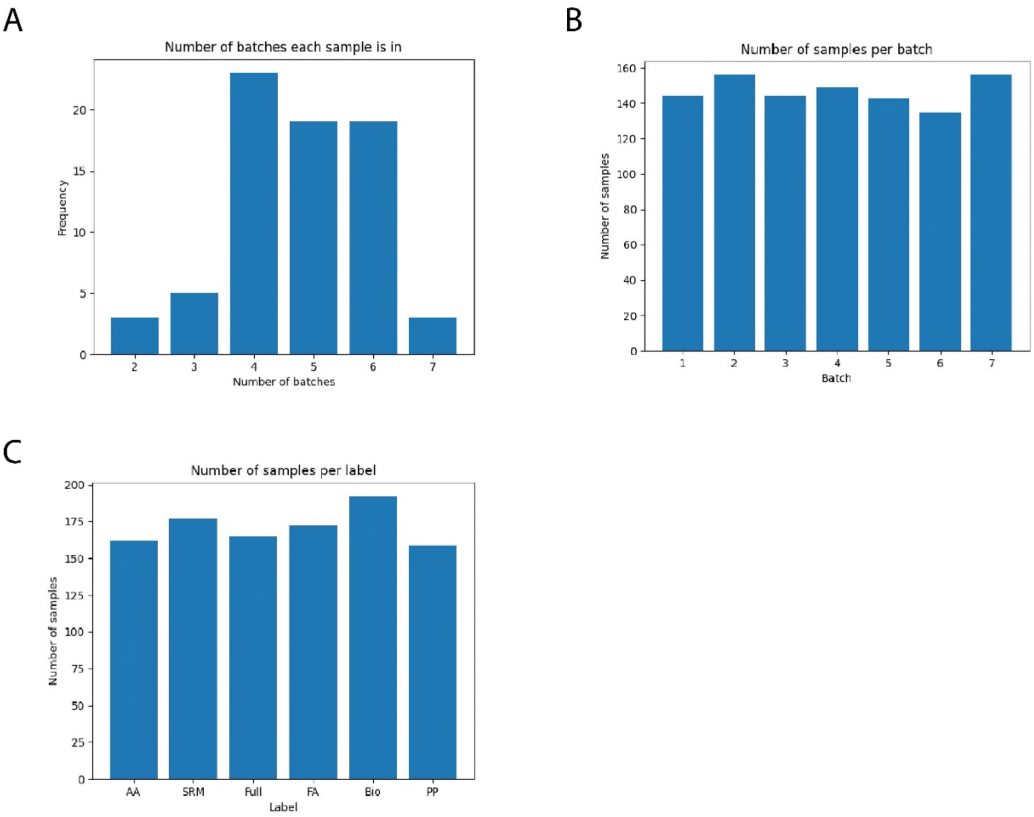

Supplementary Figure 10: Descriptive statistics of the benchmark dataset.

A) Histogram of the number of batches each sample is present. B) Number of samples per batch. C) Number of samples per class.

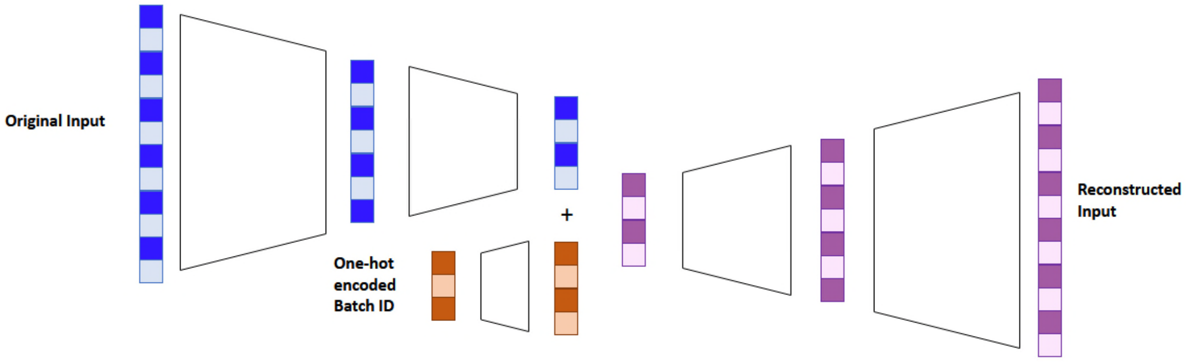

Supplementary Figure 11: Autoencoder with batch mapping.
Batch information is encoded into a vector of the same size as the bottleneck representation of the autoencoder using a single-layer neural network. The two vectors of the same size are added together before being reconstructed. This allows the reconstruction to be more accurate, because it needs the batch information to do so, but the encoded representation is trained to be free of batch information.

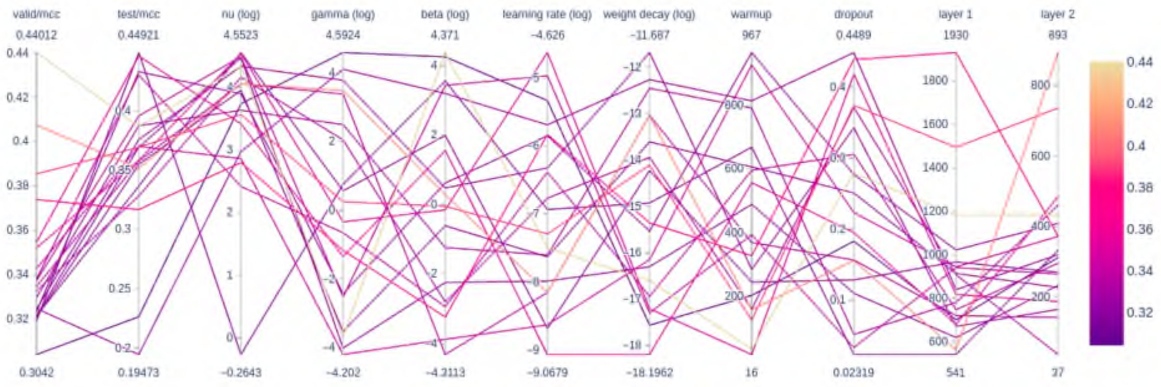

Supplementary Figure 12: Parallel coordinates plot of the hyperparameter optimization for the model VAE-DANN for the Alzheimer dataset.

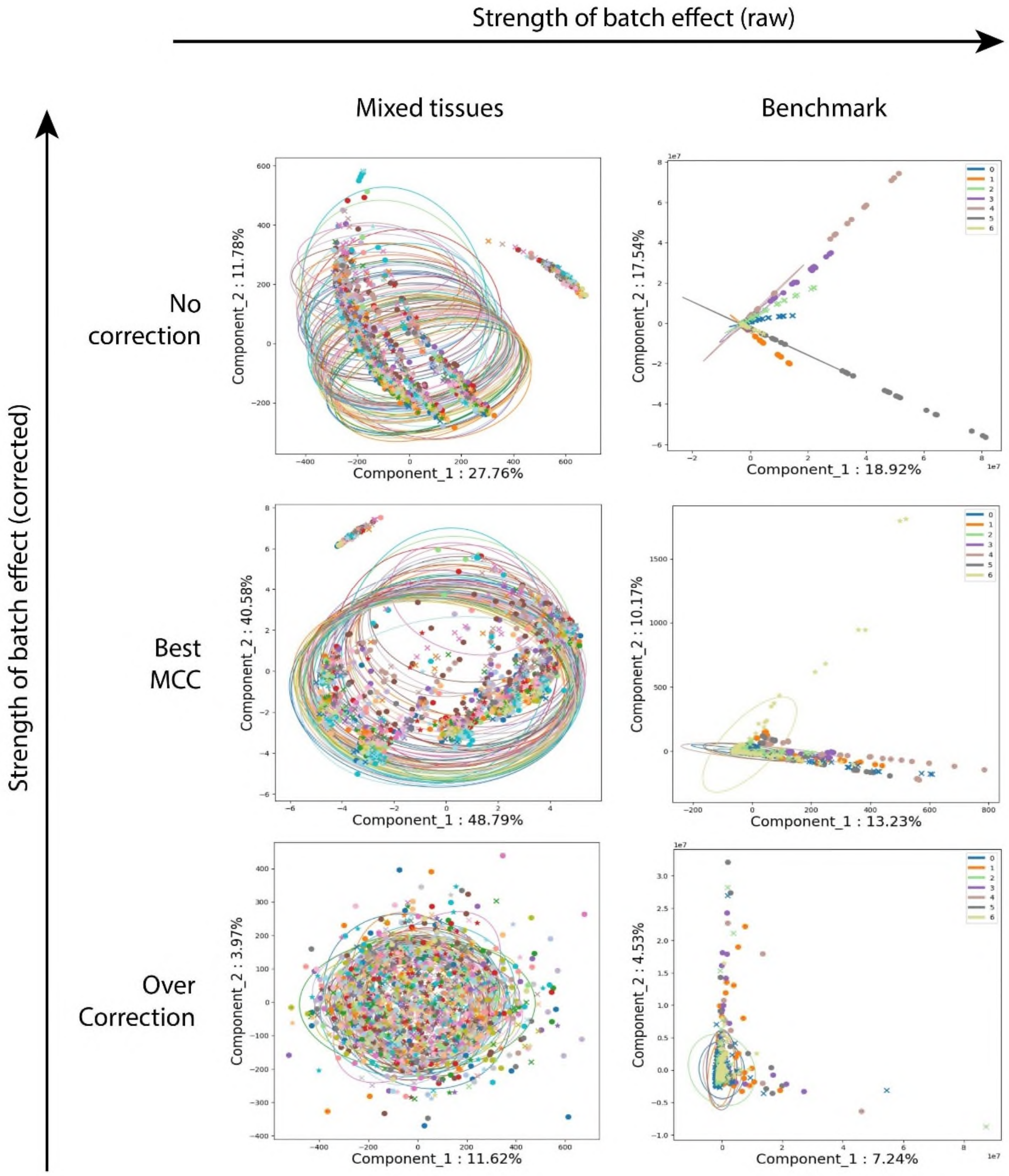

Supplementary Figure 13: PCA Visualization of the raw data for the two datasets not represented in Figure 2. The datasets are, from left to right, ordered by strength of the initial batch effect. For each dataset, the middle row of images represents the transformation resulting in the best valid MCC. The images in the last row are from representations that were in the top methods purely for batch correction but performed badly for classification.

Supplementary Table 1 – Batch effect correction metrics summary tables for the Alzheimer dataset. Source data are provided as a Source Data file.

Model name	Valid MCC	nBE	ARI	AMI	QC nMED	QC aPCC
raw	0.14	0.35	0.04	0.13	1.05	0.65
minmax	0.18	0.36	0.04	0.17	0.96	0.55
minmax_per_batch	0.22	0.34	0.11	0.24	3.26	0.17
standard	0.26	0.35	0.03	0.11	0.82	0.33
standard_per_batch	0.14	0.26	0.01	0.03	3.58	0.23
robust	0.23	0.36	0.05	0.15	1.14	0.20
robust_per_batch	0.09	0.28	**0.00**	0.01	1.75	0.20
combat	0.02	0.23	**0.00**	**0.00**	0.96	0.00
pycombat	0.16	0.32	0.01	0.03	0.87	0.77
harmony	0.18	0.23	**0.00**	**0.00**	0.77	0.03
waveica	0.13	0.33	0.01	0.02	0.78	0.88
AE	0.37	0.27	0.10	0.25	0.66	0.60
normae	0.37	0.27	0.04	0.11	**0.32**	0.89
AE-DANN	0.36	0.26	0.06	0.16	0.50	0.64
AE-invTriplet	0.40	0.28	0.03	0.10	0.62	0.35
AE-revTriplet	0.39	0.25	0.01	0.04	0.44	0.35
VAE	0.39	0.21	0.08	0.18	1.21	0.91
normvae	0.39	**0.15**	0.04	0.09	0.73	0.21
**VAE-DANN**	**0.44**	0.18	0.05	0.12	0.55	**0.99**
VAE-invTriplet	0.42	**0.15**	0.03	0.07	**0.32**	0.71
VAE-revTriplet	0.38	0.18	0.05	0.11	0.68	0.96

Supplementary Table 2 – Batch effect correction metrics summary tables for the Adenocarcinoma dataset. Source data are provided as a Source Data file.

Model name	Valid MCC	nBE	ARI	AMI	QC nMED	1 - QC aPCC
raw	0.13	0.95	1.00	1.00	0.84	0.82
minmax	0.08	0.98	1.00	1.00	0.90	0.48
minmax_per_batch	0.00	0.88	1.00	1.00	0.97	0.33
standard	0.12	0.98	1.00	1.00	0.91	0.18
standard_per_batch	0.09	0.67	0.94	0.91	0.96	0.24
robust	0.11	0.99	1.00	1.00	0.88	0.31
robust_per_batch	0.13	0.56	0.74	0.70	1.04	0.36
combat	0.11	0.43	**0.00**	**0.00**	1.18	0.01
pycombat	0.06	0.86	0.08	0.27	0.79	0.94
harmony	0.17	**0.11**	0.99	0.99	1.00	0.18
waveica	0.21	0.79	0.07	0.14	0.68	**0.98**
AE	0.21	0.51	0.94	**0.00**	0.73	0.23
normae	0.19	0.50	0.68	**0.00**	0.74	0.40
AE-DANN	0.19	0.42	0.46	**0.00**	**0.66**	0.47
**AE-invTriplet**	**0.24**	0.54	0.83	**0.00**	0.80	0.26
AE-revTriplet	0.15	0.33	0.21	**0.00**	0.97	0.04
VAE	0.15	0.37	0.35	**0.00**	0.68	0.58
normvae	0.16	0.38	0.27	**0.00**	0.91	0.18
VAE-DANN	0.12	0.37	0.30	**0.00**	0.70	0.40
VAE-invTriplet	0.11	0.37	0.39	**0.00**	0.79	0.32
VAE-revTriplet	0.13	0.18	0.18	**0.00**	0.77	0.46

Supplementary Table 3 – Batch effect correction metrics summary tables for the Aging Mice dataset. Source data are provided as a Source Data file.

Model name	Valid MCC	nBE	ARI	AMI
raw	0.92	0.88	0.78	0.82
minmax	0.94	0.88	0.81	0.84
minmax_per_batch	0.93	0.87	0.75	0.79
standard	0.92	0.81	0.74	0.78
standard_per_batch	0.92	0.40	0.01	0.05
robust	0.89	0.73	0.26	0.52
robust_per_batch	0.83	0.75	**0.00**	0.01
combat	0.05	0.23	**0.00**	**0.00**
pycombat	0.92	0.35	**0.00**	**0.00**
harmony	0.13	**0.07**	0.05	0.08
waveica	0.90	0.76	**0.00**	**0.00**
**AE**	**0.98**	0.55	0.62	0.68
**normae**	**0.98**	0.46	0.47	0.48
AE-DANN	0.97	0.57	0.52	0.57
**AE-invTriplet**	**0.98**	0.49	0.47	0.51
AE-revTriplet	0.97	0.29	0.13	0.15
**VAE**	**0.98**	0.32	0.23	0.25
normvae	0.97	0.18	0.04	0.06
**VAE-DANN**	**0.98**	0.19	0.06	0.06
**VAE-invTriplet**	**0.98**	0.19	0.08	0.07
**VAE-revTriplet**	**0.98**	0.16	0.08	0.09

Supplementary Table 4 – Batch effect correction metrics summary tables for the Benchmark dataset. Source data are provided as a Source Data file.

Model name	Valid MCC	nBE	ARI	AMI
raw	0.55	0.95	0.95	0.95
minmax	0.55	0.98	1.00	1.00
minmax_per_batch	0.56	0.88	0.98	0.98
standard	0.54	0.98	1.00	1.00
standard_per_batch	0.57	0.67	0.95	0.94
robust	0.53	0.99	1.00	1.00
robust_per_batch	0.65	0.56	0.95	0.95
combat	0.03	0.43	**0.05**	**0.11**
pycombat	0.37	0.86	0.91	0.91
harmony	0.01	**0.11**	0.20	0.27
waveica	0.44	0.79	0.82	0.82
AE	0.72	0.51	0.67	0.69
**normae**	**0.75**	0.50	0.76	0.76
AE-DANN	0.70	0.42	0.64	0.64
**AE-invTriplet**	**0.75**	0.54	0.75	0.75
AE-revTriplet	0.73	0.33	0.27	0.32
VAE	0.71	0.37	0.31	0.37
normvae	0.72	0.38	0.29	0.36
VAE-DANN	0.72	0.37	0.27	0.34
VAE-invTriplet	0.72	0.37	0.28	0.35
VAE-revTriplet	0.71	0.18	0.11	0.13

Supplementary Table 5 – Batch effect correction metrics summary tables for the mixed tissues dataset. Source data are provided as a Source Data file.

Model name	Valid MCC	nBE	ARI	AMI
raw	0.99	1.02	0.18	0.42
**minmax**	**1.00**	1.02	0.18	0.43
minmax_per_batch	0.99	1.09	0.24	0.54
standard	0.99	1.02	0.19	0.43
standard_per_batch	0.99	0.52	0.03	0.15
robust	0.99	0.90	0.14	0.36
robust_per_batch	0.99	0.81	**0.00**	0.09
combat	0.01	**0.31**	**0.00**	**0.00**
pycombat	0.94	1.01	0.01	0.06
harmony	0.03	2.18	0.49	0.71
waveica	0.91	1.02	**0.00**	0.05
AE	0.99	0.50	-0.04	0.06
normae	0.99	0.54	-0.02	0.05
AE-DANN	0.95	0.42	-0.02	0.03
AE-invTriplet	0.99	0.40	-0.04	0.04
AE-revTriplet	0.99	0.38	-0.04	0.04
**VAE**	**1.00**	0.38	-0.04	0.03
**normvae**	**1.00**	0.37	-0.02	0.02
**VAE-DANN**	**1.00**	0.37	-0.02	0.02
**VAE-invTriplet**	**1.00**	0.40	-0.04	0.03
**VAE-revTriplet**	**1.00**	0.40	-0.04	0.03

Supplementary Table 6 – Hyperparameters description

Hyperparameter	Type	Description
Learning rate	Float	Determinates the step size at which a model adjusts its parameters during training to minimize the loss function
Weight decay rate	Float	Weight decay is a regularization technique that adds a penalty term to the loss function, encouraging the model to have smaller weights, which helps prevent overfitting. The rate of weight decay controls the importance of weight decay.
Warmup epochs	Int	Number of epochs the non-supervised parts of the model (step 1 in Figure 1) is trained before starting to train the supervised part of the model (step 2 in Figure 1)
Dropout	Float	Number of neurons that are randomly dropped out. 0.0 <= thres < 1.0
Smoothing	Float	Label smoothing replaces one-hot encoded label vector y_hot with a mixture of y_hot and the uniform distribution: y_ls = (1 - α) * y_hot + α / K
Margin	Float	Margin for the triplet loss
Gamma	Float	Controls the importance given to the batches adversarial loss in the total loss (Methods section 5.4.5)
Beta	Float	Controls the importance given to the Kullback-Leibler loss in the total loss (Methods section 5.4.5)
Nu	Float	Controls the importance given to the classification loss in the total loss (Methods section 5.4.5)
Layer1	Int	The number of neurons in the first hidden layer of the encoder and the last hidden layer of the decoder
Layer2	Int	The number of neurons in the second hidden layer (Bottleneck)
Scaler	Categorical	Choose between ['none', 'minmax', 'robust', 'standard']

Supplementary methods

1.1. Reverse Triplet Loss

The equation of the Reverse Triplet Loss is the same as the normal Triplet loss:

$$L_{revTriplet}(A, P, N) = max(/f(A) - f(P)/_2 - /f(A) - f(N)/_2 + \alpha, 0)(SEQEquationARABIC10)$$

Where A is the anchor input, P is any Positive input of the same batch as A, N is any negative sample of a different batch than A, α is the margin between positive and negative pairs and f is the embedding given by passing the inputs through the encoder of the autoencoder. Using this equation would result in samples from the same batch to cluster together and different batches to be far away from each other. The distance between the clusters is controlled by the hyperparameter α. The Reverse Triplet loss does the opposite by applying a GRL for each embedding in the equation, which encourages batch-free representations. The samples from different batches get closer, while samples from the same batch are pushed further apart. The latter objective is used to prevent all samples from collapsing. If the samples from the same batch are not pushed apart, the loss would be optimal if all samples were transformed into the exact same value, which is not the desired outcome. The distance minimized in this case is the Euclidean distance, but any distance could be used.
