# Supplementary material for: BERNN: Enhancing classification of Liquid Chromatography Mass Spectrometry data with batch effect removal neural networks
Source: Nat Commun. 2024 May 6;15:3777. doi: 10.1038/s41467-024-48177-5 (PMC11074280; doi:10.1038/s41467-024-48177-5)
Supplement: Supplementary file 3 — Description of Additional Supplementary Files [file 41467_2024_48177_MOESM3_ESM.pdf]

File Name: Supplementary Data 1

Description: Contains the list of known contaminant genes
